# Tremor as an intrinsic feature of juvenile myoclonic epilepsy

**DOI:** 10.1111/epi.18268

**Published:** 2025-01-16

**Authors:** Alessia Giugno, Francesco Fortunato, Ilaria Sammarra, Miriam Sturniolo, Enrico Fratto, Iolanda Martino, Rita Nisticò, Antonio Gambardella

**Affiliations:** ^1^ Department of Medical Sciences, Institute of Neurology Magna Græcia University Catanzaro Italy; ^2^ Department of Medical and Surgical Sciences, Neuroscience Research Center Magna Græcia University Catanzaro Italy

**Keywords:** cortical hyperexcitability, drug resistance, juvenile myoclonic epilepsy (JME), tremor

## Abstract

We aim to understand whether tremor may be an intrinsic feature of juvenile myoclonic epilepsy (JME) and whether individuals with JME plus tremor experience a different disease course. Thirty‐one individuals with JME plus tremor (17 females, mean age = 33.9 ± 13.8 years) and 30 age of onset‐ and gender‐matched subjects with JME (21 females, mean age = 26.8 ± 11.2 years) prospectively underwent clinical and neurophysiologic assessment, including tremor assessment and somatosensory evoked potentials (SEPs). All JME plus tremor subjects experienced postural and action tremor affecting bilateral upper limbs. Nine of 31 individuals (29%) with tremor were never exposed to valproate (VPA), and 14 of 31 (45.2%) were not using VPA at the time of clinical evaluation. Twelve of 31 (38.7%) patients with JME plus tremor were drug‐resistant compared to four of 30 (13.3%) with JME (*p* = .024). The JME plus tremor subjects had higher numbers of previous childhood absence epilepsy (*n* = 6/31 [19.4%]), interictal epileptiform discharges (*n* = 30/31 [96.8%]), photosensitivity (*n* = 8/31 [25.8%]), and psychiatric comorbidities (*n* = 12/31 [38.7%]). Six of 31 (19.4%) individuals with JME plus tremor had giant SEPs (1/30, 3.3% with JME; *p* = .05, chi‐squared test). The clinical features and decreased sensorimotor inhibition in the JME plus tremor group suggest that tremor might be a marker of disease severity rather than an epiphenomenon of VPA exposure.

## 1 INTRODUCTION

Juvenile myoclonic epilepsy (JME) is the most common presumed genetic generalized epileptic syndrome, characterized by juvenile onset of myoclonic, generalized tonic–clonic seizures (GTCS) and, less often, absences.^1^, ^2^, ^3^

Neurological examination in JME is usually unremarkable; however, some prospective series have reported hand tremor in up to 35% of individuals with JME.^4^ Tremor in JME is usually attributed antiseizure medications (ASMs), such as valproate (VPA).^5^ Nevertheless, preliminary evidence suggest that tremor may also occur independently from drug exposure.^6^ In a subgroup of individuals with JME and tremor, tremor has been demonstrated to occur independently from drugs with electrophysiological signatures of cortical hyperexcitability, such as enhanced cortical amplitudes in somatosensory evoked potentials (SEPs).^6^

Juvenile myoclonic epilepsy is also widely recognized as a constellation of different phenotypes^2^ with some clear electroclinical markers of drug resistance, such as absence seizures, photoparoxysmal response, stress‐related factors, and psychiatric comorbidities.^7^, ^8^ It is largely unknown whether JME patients with tremor may be more pharmacoresistant to ASMs.

In this study, we aimed to address the following questions: (1) Can tremor in JME be part of the syndrome itself, or is it always a side effect of medications? (2) Do individuals with JME and tremor experience a different disease course (e.g., drug resistance) compared to those with the classic phenotype? (3) Are there distinct electrophysiological signatures between these two subgroups?

## 2 MATERIALS AND METHODS

### 2.1 Cohort selection

From October 2021 until May 2024, we prospectively enrolled subjects diagnosed with JME, aged 18 years or older, at the Outpatient Epilepsy Clinic at Magna Græcia University in Catanzaro, Italy. The study was approved by the institution's ethical committee and conducted in accordance with the Helsinki Declaration and its later amendments. All individuals provided written informed consent.

At the time of the clinical evaluation, detailed phenotyping was performed for each individual, including family history of epileptic and/or febrile seizures, age at onset of the first epileptic seizure, type of the first seizure, seizure types throughout disease course, status epilepticus throughout the disease course, history of previous childhood absence epilepsy (CAE), current ASMs with daily dosage and plasma level monitoring, start date and maximum dosage of VPA, drug resistance per International League Against Epilepsy (ILAE) criteria,^9^ and psychiatric comorbidities per Diagnostic and Statistical Manual of Mental Disorders, 5th edition criteria.^10^ Two trained neurologists (A.Gi. and F.F.) conducted a neurological examination on all subjects with a specific focus on tremor, according to Movement Disorder Society criteria.^11^ Additionally, two neurologists (I.S. and E.F.), blinded to the clinical evaluations, retrospectively reviewed the clinical records of each patient to identify any tremor documented and to check the onset of the tremor.

In addition, each patient underwent standard electroencephalogram (EEG) recording with hyperventilation (HV) and photic stimulation according to the latest ILAE recommendations^12^ and 3‐T brain magnetic resonance imaging to exclude other structural causes of epilepsy. EEGs were classified as either normal or showing interictal epileptiform discharges (IEDs) such as generalized 3–5.5‐Hz spike–wave or polyspike‐wave activity. We categorized HV as either absence or presence of evoked IEDs and photostimulation as either normal or showing a photoparoxysmal response.

The diagnosis of JME has been made according to criteria.^1^ Alert and exclusion criteria were aligned with those of the ILAE.^1^ For subjects with previous CAE, we considered the age at first myoclonic or GTCS as seizure onset. To prevent confounding with familial adult myoclonic epilepsy (FAME),^13^ we excluded participants with a history of autosomal dominant inheritance of tremor and/or epileptic seizures.

Other exclusion criteria included poor ASM compliance, limb neuropathy or myelopathy, tremor associated with bradykinesia, rigidity, and dystonic features.

We categorized participants with JME who exhibited subtle rhythmic postural tremor as the “JME plus tremor” cohort and those without tremor as "JME." For each subject in the JME plus tremor cohort, an age at onset‐ and sex‐matched control with JME but without tremor was enrolled.

### 2.2 Electrophysiological assessment of tremor and SEP recordings

All patients underwent tremor assessment, as previously reported.^14^ Hand tremor has been characterized in terms of amplitude, frequency, and pattern.^14^ The SEP recording protocol can be found in Data S1. N20–P25 normative values (in microvolts) calculated from 200 healthily controls can be found in Data S2.

### 2.3 Statistical analysis

Statistical analysis was conducted using JASP statistical software (version 0.19.0).^15^ The sample size calculation for this case–control study was based on G*power 3 tool.^16^ The calculation was based on a desired power of .80 and an alpha level of .05. Our analysis indicated that a sample size of 61 participants would be able to identify medium effect (effect size = .4) of the variables of interest. Shapiro–Wilk test verified the normal distribution of each variable. Data were normally distributed for *p*‐values of >.05. Chi‐squared or Fisher exact test, as appropriate, for binary or categorical variables, and *t*‐test for continuous variables, were used for association tests. The nonparametric Mann–Whitney test compared data with nonnormal distribution. Analysis of covariance with Bonferroni post hoc analysis assessed SEP latencies and amplitudes between JME plus tremor and JME cohorts, using height and age as covariates. A *p*‐value of <.05 was considered significant after correction for multiple comparisons using the Bonferroni method.

## 3 RESULTS

### 3.1 JME cohort description

We definitively recruited 31 individuals belonging to the JME plus tremor group (17 females, mean age = 33.9 ± 13.8 years) and 30 age at onset‐ and gender‐matched controls with JME (21 females, mean age = 26.8 ± 11.2). Comprehensive demographic and clinical features are reported in Table 1. Additional details on our cohort are reported in Data S1.

**TABLE 1:** Demographic and clinical features of subjects with JME and JME plus tremor.

Feature	JME, *n* = 30	JME plus tremor, *n* = 31	*p*	Bonferroni correction
Gender, *n* (female %)	21 (70%)	17 (54.8%)	.222^a^	ns
Height, cm^b^	166.9 ± 7.4	168.2 ± 9.4	.879^c^	ns
Age at time of observation, years^b^	26.8 ± 11.2	33.9 ± 13.8	.034^c^	ns
Age at epilepsy onset, years^b^	14.5 ± 4.7	14.1 ± 4.8	.572^c^	ns
Age at tremor onset, years^b^	‐	14.2 ± 6.7	‐	
Age at confirmed JME diagnosis, years^b^	15 ± 4.6	14.7 ± 4.8	.647^c^	ns
Disease duration, years^b^	12.3 ± 11.4	19.9 ± 13.7	.02^c^	ns
Follow‐up from confirmed JME diagnosis, years^b^	11.8 ± 11.2	19.2 ± 13.6	.017^c^	ns
Previous CAE, *n* (%)	3 (10%)	6 (19.4%)	.303^a^	ns
Type of seizure at onset, *n* (%)				ns
Myoclonic seizures	18 (60%)	19 (61.3%)	.447^a^	
GTCS	9 (30%)	6 (19.4%)		
Absence seizures	3 (10%)	6 (19.4%)		
Seizures, *n* (%)				ns
Myclonic + GTCS	25 (83.3%)	29 (93.6%)	.211^a^	
Myoclonic + absence seizures	4 (13.3%)	8 (25.8%)		
Spike and wave discharges on EEG	25 (83.3%)	30 (96.8%)	.078^a^	ns
Photosensitive trait on EEG, *n* (%)	6 (20%)	8 (25.8%)	.590^a^	ns
ASMs, *n* (%)				ns
No ASMs	0 (0%)	2 (6.4%)	.130^a^	
Monotherapy	23 (80%)	16 (51.6%)		
Polytherapy	7 (23.3%)	13 (41.9%)		
VPA at clinical evaluation, *n* (%)	8 (26.7%)	14 (45.2%)	.133^a^	ns
Drug‐refractory epilepsy, *n* (%)	4 (13.3%)	12 (38.7%)	.024^a^	ns
Psychiatric comorbidities, *n* (%)	10 (33.3%)	12 (38.7%)	.662^a^	ns

Abbreviations: ASM, antiseizure medication; CAE, childhood absence epilepsy; EEG, electroencephalogram; GTCS, generalized tonic–clonic seizures; JME, juvenile myoclonic epilepsy; ns, not significant; VPA, valproate.

^a^ Probability value after chi‐squared test.

^b^ Data are expressed as mean ± SD.

^c^ Probability value after Mann–Whitney test.

### 3.2 Tremor assessment and VPA exposure in JME plus tremor

All participants belonging to the JME plus tremor cohort experienced a postural and action tremor affecting bilateral upper limbs. No resting component or reemergent tremor has been identified. The mean frequency of the tremor was 8.05 ± 1.56 Hz. Nine of 31 individuals (29%) were not exposed to VPA during the disease course. Twenty‐two of 31 (70.97%) had VPA exposure during disease course, with only 14 of 31 (45.2%) using VPA at the time of clinical evaluation. Interestingly, 13 of 22 (59.1%) individuals had tremor onset reported in clinical charts before initiation of VPA. For nine of 22 (40.1%) patients, tremor was reported as concomitant with or later than VPA exposure.

The number of people treated with VPA (*p* = .133, chi‐squared test) and daily dosage of VPA (*p* = .722) were not different between the two groups.

### 3.3 Clinical features of JME plus tremor group

At the time of clinical evaluation, 12 of 31 (38.7%) of people with JME plus tremor were drug‐resistant compared to four of 30 (13.3%) in the JME group (*p* = .024, chi‐squared test). There were other clinical features in JME plus tremor indicating a more severe disease compared to JME, such as the number of individuals with a previous diagnosis of CAE (*n* = 6/31 [19.4%]), IEDs on EEG (*n* = 30/31 [96.8%]), photosensitivity (*n* = 8/31 [25.8%]), and psychiatric comorbidities (*n* = 12/31 [38.7%]). None of the enrolled subjects had IEDs related to HV.

However, none of these clinical differences survived after multiple comparison correction.

At the time of observation, ASMs in the JME plus tremor cohort were distributed as follows: 16 of 31 (51.6%) were on monotherapy, 13 of 31 (41.9%) on polytherapy, and two of 31 (6.4%) were without any ASM. The three ASMs most prescribed for both groups were (1) levetiracetam (LEV), (2) VPA, and (3) lamotrigine (LTG), with no different distribution between the two groups. At the time of clinical evaluation, serum blood levels of ASMs were all within normal laboratory ranges. Further details regarding ASMs and their daily dosages are summarized in Data S1.

### 3.4 Analysis of SEPs

Data on SEPs recordings are shown in Table 2. N9 and N13 latencies did not differ between the two study groups, so we can reasonably exclude a comparison bias due to a defect in peripheral conduction. Six of 31 (16.1%) individuals in the JME plus tremor cohort had giant SEPs compared to one of 30 (3.3%) in the JME cohort (*p* = .05, chi‐squared test). No one from either group experienced an enhanced long‐loop reflex. The comprehensive analysis of SEPs also revealed some interesting trends, but these were not statistically significant (for further details, please see Table 2).

**TABLE 2:** SEP recordings in JME and JME plus tremor cohorts.

	JME, *n* = 30	JME plus tremor, *n* = 31	*p* < .05 (Bonferroni threshold = .005)
N9, ms, median [IQR]			
Left	9.15 [.68]	9.70 [.85]	.435^a^
Right	9.25 [.80]	9.70 [1.10]	.435^a^
N13, ms, median [IQR]			
Left	12.65 [1.00]	12.95 [1.05]	.125^a^
Right	12.85 [1.00]	13.50 [1.30]	.194^a^
N20, ms, median [IQR]			
Left	18.15 [1.40]	19.50 [1.75]	.005^a^
Right	18.25 [1.30]	19.60 [2.25]	.008^a^
N20–N13, ms, median [IQR]			
Left	5.50 [1.48]	5.70 [1.70]	.817^a^
Right	5.30 [.78]	5.80 [1.60]	.579^a^
P22, ms, median [IQR]			
Left	20.75 [1.63]	22.10 [1.85]	.013^a^
Right	21.05 [1.25]	22.10 [2.15]	.002^a^
P25, ms, median [IQR]			
Left	24.65 [1.95]	25.40 [2.30]	.277^a^
Right	25.15 [1.95]	25.80 [2.10]	.176^a^
N20–P25, μV, median [IQR]			
Left	3.10 [2.05]	4.70 [5.05]	.003^a^ ^,^ *
Right	3.20 [3.418]	4.90 [3.95]	.029^a^
N20–P22, μV, median [IQR]			
Left	3.90 [2.70]	4.10 [2.70]	.284^a^
Right	3.80 [2.85]	3.30 [1.65]	.969^a^
Giant SEPs, *n* (%)	1 (3.3%)	6 (19.4%)	.05^b^

Abbreviations: IQR, interquartile range; JME, juvenile myoclonic epilepsy; SEP, somatosensory evoked potential.

^a^ Analysis of covariance with Bonferroni test using height and gender as covariates.

^b^ Chi‐squared test.

* Probability value significant after multiple comparison correction.

## 4 DISCUSSION

To our knowledge, this is the most comprehensive study exploring tremor features in JME. In literature, there remains uncertainty about whether tremor reflects VPA exposure or a different underlying pathophysiology.^6^

Our findings demonstrated that, at least in a subset of individuals, tremor may be an intrinsic feature of JME rather than a mere epiphenomenon of drug exposure. We have shown that nine of 31 (29%) individuals with JME plus tremor had no previous or ongoing VPA exposure. Of course, we cannot entirely rule out the contribution of the other ASMs to the development of tremor, because tremor has been reported as a side effect of LTG in up to 10% of users.^17^ Additionally, there are reports associating tremor development with LEV exposure.^18^ In our study, only seven of 31 (22.6%) individuals in the JME plus tremor group were on LTG at the time of clinical evaluation, and both the JME plus tremor and JME groups were quite balanced in terms of global ASM regimen (i.e., number of people exposed to LTG or LEV) as well as for daily dosages (in milligrams). Additionally, we checked serum blood levels of ASMs at the time of clinical observation, and they were all in normal range. It is also insightful to note that, as reported by Sisodiya's group,^19^ tremor has only been anecdotally associated with other ASMs.^19^ Furthermore, 13 of 22 (59.1%) individuals who had previous/concurrent VPA exposure manifested tremor before using VPA, as documented by clinical records. In addition, differently from VPA‐induced tremor, our patients presented high‐frequency postural tremor, with superimposed myoclonic components and no rest tremor.^20^

The clinical characteristics of the JME plus tremor group in our cohort suggest that tremor may be a marker of more severe disease in JME. In patients with tremor, we observed greater numbers with prior CAE, abnormal routine EEG findings, photosensitivity, and psychiatric comorbidities, as well as a higher median number of ASMs. Moreover, we observed a high proportion of drug resistance (38.7%)^8^ in the JME plus tremor group, compared to 13.3% in the JME cohort. None of those enrolled in the resistant group reported poor compliance, and ASMs serum concentrations were all within normal range. Thus, we can reasonably rule out noncompliance in the JME drug‐resistant cohort.

Finally, electrophysiological studies further support our clinical observations. Specifically, we found a higher proportion of giant SEPs in the JME plus tremor compared to the JME group. Our extensive neurophysiological assessment suggests decreased inhibition of the sensorimotor cortex, associated with more severe frontothalamic dysfunction in the presence of tremor. The JME plus tremor group presented increased SEP cortical latencies and amplitudes. The prolonged N20 and P22 latencies and the increased N20–P25 amplitudes reflect the activation of somatosensory cortex, receiving in turn inputs from thalamus. Both neurophysiological and neuroimaging studies extensively illustrated the aberrant thalamic excitability and frontothalamic circuitry in JME, which are involved in generating spike–wave complexes and myoclonic seizures.^21^

Our study has several limitations. First, we did not perform genetic testing for intronic expansions to rule out a diagnosis of FAME in the JME plus tremor group. However, the absence of autosomal dominant inheritance of tremor and/or seizures reduces the likelihood of this confounding factor. Additionally, the seizure semiology of our cohort, characterized by prominent myoclonic seizures along with tremor, does not align with the classical FAME phenotype. Another important limitation of not performing genetic testing is that we cannot exclude the possibility that some individuals may present with a JME phenotype of a progressive myoclonic epilepsy, such as Unverricht–Lundborg disease. However, we previously demonstrated a very low rate of *CSTB* expansions in individuals with JME phenotype.^22^

We strongly encourage clinicians to assess for tremor in individuals with JME, as it may be a core feature of the syndrome and a marker for a more severe disease course. Further studies may explore genetics in the JME plus tremor group to identify potential differences compared to the pure JME phenotype.

## AUTHOR CONTRIBUTIONS

**Alessia Giugno:** Conceptualization; data curation; formal analysis; investigation; methodology; writing—original draft preparation. **Francesco Fortunato:** Conceptualization; data curation; formal analysis; investigation; methodology; supervision; writing—review and editing. **Ilaria Sammarra:** Data curation; formal analysis; investigation. **Miriam Sturniolo:** Data curation; methodology. **Enrico Fratto:** Data curation; formal analysis; investigation. **Iolanda Martino:** Data curation; investigation. **Rita Nisticò:** Data curation; investigation. **Antonio Gambardella:** Conceptualization; data curation; investigation; methodology; supervision; writing—review and editing; funding acquisition.

## FUNDING INFORMATION

This work was supported by #NEXTGENERATIONEU and funded by the Ministry of University and Research, National Recovery and Resilience Plan, project MNESYS (PE0000006)—A Multiscale Integrated Approach to the Study of the Nervous System in Health and Disease (DN 1553 11.10.2022).

## CONFLICT OF INTEREST STATEMENT

None of the authors has any conflict of interest to disclose. We confirm that we have read the Journal's position on issues involved in ethical publication and affirm that this report is consistent with those guidelines.

## ETHICS STATEMENT

This survey has been approved by our institution's ethics committee and has been performed in accordance with the ethical standards laid down in the 1964 Declaration of Helsinki and its later amendments. We obtained patients' informed consent, and data were treated according to the European regulation GDPR n. 2016/679.

## Supporting information


Data S1.

Data S2.

## Data Availability

Deidentified data that support the findings of this study are available upon request to the corresponding author.

## References

[1] Hirsch E , French J , Scheffer IE , Bogacz A , Alsaadi T , Sperling MR , et al. ILAE definition of the Idiopathic Generalized Epilepsy Syndromes: position statement by the ILAE Task Force on Nosology and Definitions. Epilepsia. 2022;63:1475–1499. 35503716 10.1111/epi.17236

[2] Baykan B , Wolf P . Juvenile myoclonic epilepsy as a spectrum disorder: a focused review. Seizure. 2017;49:36–41. 28544889 10.1016/j.seizure.2017.05.011

[3] Vorderwülbecke BJ , Wandschneider B , Weber Y , Holtkamp M . Genetic generalized epilepsies in adults: challenging assumptions and dogmas. Nat Rev Neurol. 2022;18:71–83. 34837042 10.1038/s41582-021-00583-9

[4] Panayiotopoulos CP , Obeid T , Tahan AR . Juvenile myoclonic epilepsy: a 5‐year prospective study. Epilepsia. 1994;35:285–296. 8156946 10.1111/j.1528-1157.1994.tb02432.x

[5] Zhang CQ , He BM , Hu ML , Sun HB . Risk of valproic acid‐related tremor: a systematic review and meta‐analysis. Front Neurol. 2020;11:576579. 33384651 10.3389/fneur.2020.576579 PMC7769765

[6] Aydin‐Özemir Z , Matur Z , Baykan B , Bilgic B , Tekturk P , Bebek N , et al. Analysis of the tremor in juvenile myoclonic epilepsy. Epilepsy Res. 2016;128:140–148. 27835783 10.1016/j.eplepsyres.2016.10.010

[7] Stevelink R , Koeleman BPC , Sander JW , Jansen FE , Braun KPJ . Refractory juvenile myoclonic epilepsy: a meta‐analysis of prevalence and risk factors. Eur J Neurol. 2019;26:856–864. 30223294 10.1111/ene.13811 PMC6586162

[8] Stevelink R , Al‐Toma D , Jansen FE , Lamberink HJ , Asadi‐Pooya AA , Farazdaghi M , et al. Individualised prediction of drug resistance and seizure recurrence after medication withdrawal in people with juvenile myoclonic epilepsy: a systematic review and individual participant data meta‐analysis. EClinicalMedicine. 2022;53:101732. 36467455 10.1016/j.eclinm.2022.101732 PMC9716332

[9] Kwan P , Arzimanoglou A , Berg AT , Brodie MJ , Allen Hauser W , Mathern G , et al. Definition of drug resistant epilepsy: consensus proposal by the ad hoc task force of the ILAE commission on therapeutic strategies. Epilepsia. 2010;51:1069–1077. 19889013 10.1111/j.1528-1167.2009.02397.x

[10] American Psychiatric Association . Diagnostic and statistical manual of mental disorders. 5th ed. Washington, DC: American Psychiatric Association; 2013.

[11] Bhatia KP , Bain P , Bajaj N , Elble RJ , Hallett M , Louis ED , et al. Consensus statement on the classification of tremors. From the task force on tremor of the International Parkinson and Movement Disorder Society. Mov Disord. 2018;33:75–87. 29193359 10.1002/mds.27121 PMC6530552

[12] Peltola ME , Leitinger M , Halford JJ , Vinayan KP , Kobayashi K , Pressler RM , et al. Routine and sleep EEG: minimum recording standards of the International Federation of Clinical Neurophysiology and the International League Against Epilepsy. Epilepsia. 2023;64:602–618. 36762397 10.1111/epi.17448 PMC10006292

[13] Ishiura H , Doi K , Mitsui J , Yoshimura J , Matsukawa MK , Fujiyama A , et al. Expansions of intronic TTTCA and TTTTA repeats in benign adult familial myoclonic epilepsy. Nat Genet. 2018;50:581–590. 29507423 10.1038/s41588-018-0067-2

[14] Nisticò R , Pirritano D , Salsone M , Novellino F , Del Giudice F , Morelli M , et al. Synchronous pattern distinguishes resting tremor associated with essential tremor from rest tremor of Parkinson's disease. Parkinsonism Relat Disord. 2011;17:30–33. 21071257 10.1016/j.parkreldis.2010.10.006

[15] JASP Team . JASP (version 0.19.0). 2024. https://jasp‐stats.org/faq/how‐do‐i‐cite‐jasp/

[16] Faul F , Erdfelder E , Lang A‐G , Buchner A . G*power 3: a flexible statistical power analysis program for the social, behavioral, and biomedical sciences. Behav Res Methods. 2007;39(2007/05/01):175–191. 17695343 10.3758/bf03193146

[17] Yang JH , Chung SW , Kim JS . Action tremor associated with lamotrigine monotherapy. J Mov Disord. 2010;3:18–19. 24868374 10.14802/jmd.10005 PMC4027652

[18] Gupta N , Seier M , Vuppala A . Tremor as a probable adverse drug reaction to levetiracetam: a case report. Grad Med Educ Res J. 2021;3:41.

[19] Silvennoinen K , de Lange N , Zagaglia S , Balestrini S , Androsova G , Wassenaar M , et al. Comparative effectiveness of antiepileptic drugs in juvenile myoclonic epilepsy. Epilepsia Open. 2019;4:420–430. 31440723 10.1002/epi4.12349 PMC6698679

[20] Paparella G , Angelini L , De Biase A , Cannavacciuolo A , Colella D , Di Bonaventura C , et al. Clinical and kinematic features of valproate‐induced tremor and differences with essential tremor. Cerebellum. 2021;20:374–383. 33200286 10.1007/s12311-020-01216-5 PMC8213593

[21] Hattingen E , Lückerath C , Pellikan S , Vronski D , Roth C , Knake S , et al. Frontal and thalamic changes of GABA concentration indicate dysfunction of thalamofrontal networks in juvenile myoclonic epilepsy. Epilepsia. 2014;55:1030–1037. 24902613 10.1111/epi.12656

[22] Mumoli L , Tarantino P , Michelucci R , Bianchi A , Labate A , Franceschetti S , et al. No evidence of a role for cystatin B gene in juvenile myoclonic epilepsy. Epilepsia. 2015;56:e40–e43. 25752200 10.1111/epi.12944

