# Supplementary material for: Tremor as an intrinsic feature of juvenile myoclonic epilepsy
Source: Epilepsia. 2025 Jan 16;66(3):e47–53. doi: 10.1111/epi.18268 (PMC11908659; doi:10.1111/epi.18268)
Supplement: Supplementary file 1 — Data S1. [file EPI-66-e47-s001.docx]

**Supplementary Materials**

**1) Supplementary material 1: Somatosensory evoked potentials (SEPs) recording protocol**

**2) Supplementary material 2: List of medications of the JME cohort and daily dosage**

**3) Supplementary Figure 1: List of medications of the JME cohort**

**4) Supplementary Table 1: Demographic and clinical features of ‘JME plus’ cohort**

**5) Supplementary Table 2: Demographic and clinical features of ‘JME’ cohort**

**6) Supplementary material 3: EEG data**

**1) Somatosensory evoked potentials (SEPs) recording protocol**

SEPs recordings were obtained in the morning between 10 a.m. and 12 p.m in a warm, semi-darkened room. SEPs were elicited by unilateral percutaneous electrical stimulation of the median nerve at wrist with intensity of 110% of the movement threshold (stimulus duration: 0.1 ms; stimulation frequency: 3.5 Hz; high-pass filter 5 Hz, low-pass filter 2 kHz; analysis time base: 5 ms/D; impedance was kept below 5000 Ω; Dantec® Keypoint®). Two sets of 1000 trials were consecutively collected, superimposed, and averaged. Automatic artefact rejection was used to eliminate occasional high-amplitude transients (> 100 μV). To record peripheral (N9 and N13) and cortical (N20, P22, P25) SEPs components, electrodes were placed as reported elsewhere11. The N9 and N13 peripheral latencies were closely monitored to rule out subjects with evidence of abnormalities. Peak amplitude of SEPs components was manually extracted from the individual waveforms. For each patient and for each side of stimuli, we analyzed N20, P22, P25 latencies (milliseconds, msec), N20-P25 and N20-P22 amplitudes (micronVolt, μV). SEPs were defined as “giant,” if peak-to-peak amplitude of N20-P25 exceed the mean value+3SD of our normative laboratory values (N20-P25 > 10.5 μV). Cortical reflex (c-reflex or long latency reflex I) was recorded by a pair of disc electrodes on both abductor pollicis brevis muscles as the. Therefore, we analyzed N22 and P25 latencies (msec) and N22-P25 amplitudes (μV).

**2) ASMs regimen of the JME plus and JME cohorts**

The ASMs were distributed in the JME plus and JME cohorts respectively as follows: 14/31 (45.2%) and 8/30 (26.7%) were treated with valproate [Chi squared test; P=0.133]; 19/31 (61.3%) and 18/30 (60%) were treated with levetiracetam [Chi squared test; P=0.918]; 7/31 (22.6%) and 5/30 (16.7%) received lamotrigine [Chi squared test; P=0.561]. As reported below in the tables 1 and 2, both JME plus and JME groups shared a similar daily dosage of valproate [(JME plus: 1003.6±413.9 milligram per day; JME: 1000 ±353.6 milligram per day; P=0.722 (Mann-Whitney test)] and levetiracetam [(JME plus: 1447.4±724.5 milligram per day; JME 1344.4±665.5 milligram per day; P=0.530 (Mann-Whitney test)]. Conversely, the dosage of lamotrigine was lower in JME plus group compared JME group [ JME plus: 135.7±69.1 milligram per day; JME: 320.0±109.6 milligram per day; P=0.017 (Mann-Whitney test)].

**3) Supplementary Figure 1: List of medications of the JME cohort**

**
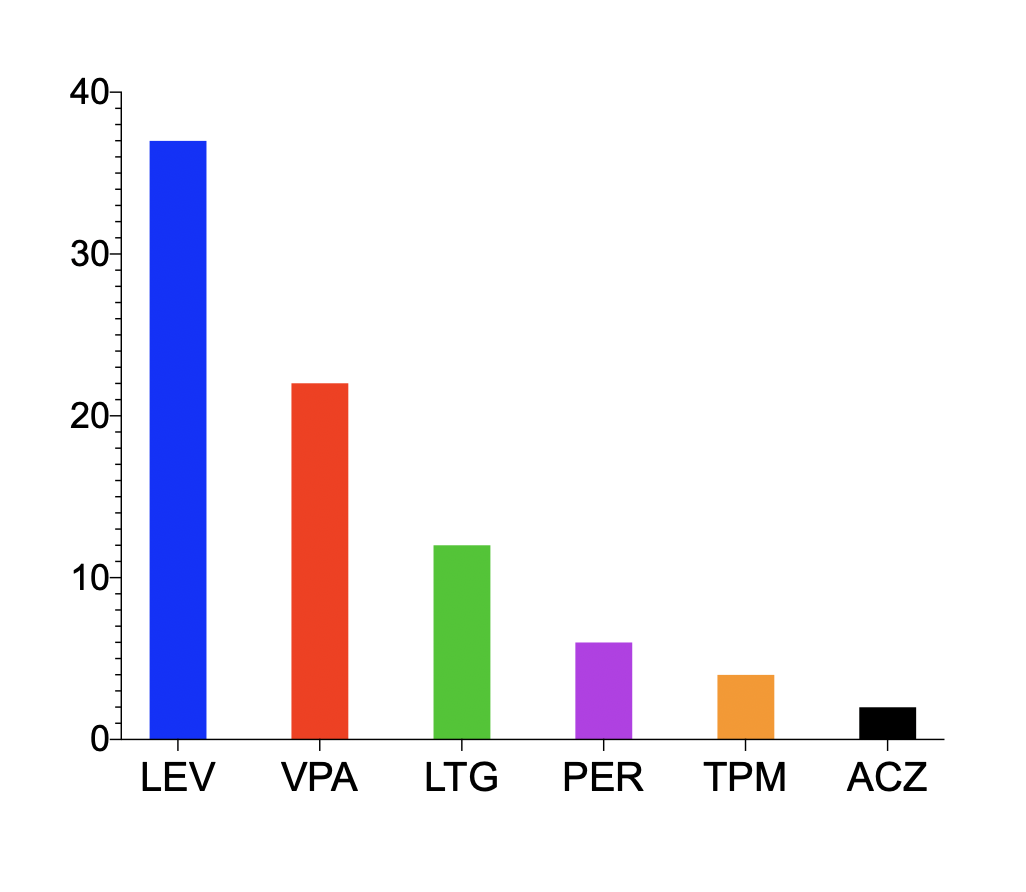
**

The y axis shows the number of each ASM per individual. **Abbreviations**: LEV= levetiracetam; VPA= valproate; LTG= lamotrigine; PER= perampanel; TPM= topiramate; ACZ= acetazolamide; LAC= lacosamide.

**4) Supplementary Table 1: Demographic and clinical features of ‘JME plus’ cohort**

| Patient | Gender | | Age | Age at JME onset  (years) | Age at tremor onset  (years) | Age at confirmed JME diagnosis | Previous CAE | Seizures during disease course | Postural hand tremor | Giant SEPs | Drug-resistant epilepsy | Ongoing ASMs  (mg/d) | Ongoing VPA |  |
| --- | --- | --- | --- | --- | --- | --- | --- | --- | --- | --- | --- | --- | --- | --- |
| 1 | | | F | 34 | 10,8 | 10,8 | 14 | No | Myoclonic and GTCS | Yes | Yes | No | TPM (150), LEV(1000) | No |
| 2 | | | F | 19 | 17 | 7 | 17 | Yes | Absences, Myoclonic, GTCS | Yes | No | No | LEV  (1500) | No |
| 3 | | | M | 31 | 8 | 7 | 12 | No | Absences, Myoclonic, GTCS | Yes | No | Yes | VPA (1250), LTG (100) | Yes |
| 4 | | | M | 60 | 15 | 15 | 16 | No | Myoclonic and GTCS | Yes | No | No | VPA (500) | Yes |
| 5 | | | F | 23 | 15,8 | 16 | 17 | No | Myoclonic, GTCS | Yes | No | No | LEV (1500) | No |
| 6 | | | F | 51 | 16 | 16 | 17 | No | Myoclonic and GTCS | Yes | No | No | VPA (800), LTG (200) | Yes |
| 7 | | | F | 39 | 14 | 14 | 14 | No | Myoclonic and GTCS | Yes | No | No | LEV (2000) , LTG (150) | No |
| 8 | | M | 31 | 15 | 15 | 15,5 | No | Myoclonic and GTCS | Yes | No | Yes | VPA (2000), TPM (50) | Yes |  |
| 9 | | M | 23 | 16 | 15 | 16 | No | Myoclonic and GTCS | Yes | No | Yes | LEV (2000),  VPA (1500)LTG (50) | Yes |  |
| 10 | | F | 14 | 13 | 5,4 | 13 | Yes | Absences, Myoclonic, GTCS | Yes | No | No | LEV (1000) | No |  |
| 11 | | F | 49 | 9 | 9 | 11 | No | Myoclonic and GTCS | Yes | No | Yes | LEV (1250), LTG (200), ACZ (300) | No |  |
| 12 | | F | 45 | 33 | 37 | 34 | Yes | Absences, Myoclonic, GTCS | Yes | No | No | VPA (500) | Yes |  |
| 13 | | F | 35 | 20 | 33 | 21 | Yes | Absences, Myoclonic, GTCS | Yes | No | No | LEV (3000) | No |  |
| 14 | | F | 18 | 8 | 12 | 8 | Yes | Absences, Myoclonic, GTCS | Yes | Yes | No | LEV (1250) | No |  |
| 15 | | M | 18 | 16 | 16 | 17 | No | Myoclonic and GTCS | Yes | Yes | No | LEV (1250) | No |  |
| 16 | | M | 34 | 13 | 13 | 14 | No | Myoclonic and GTCS | Yes | No | Yes | VPA (1000), LTG (200) | Yes |  |
| 17 | | F | 42 | 20 | 20 | 21 | No | Myoclonic and GTCS | Yes | No | No | - | No |  |
| 18 | | F | 26 | 12 | 12 | 13 | No | Myoclonic and GTCS | Yes | No | Yes | LEV (2000) | No |  |
| 19 | | M | 20 | 13 | 13 | 13 | No | Myoclonic and GTCS | Yes | No | No | - | No |  |
| 20 | | M | 48 | 14 | 17 | 14 | No | Myoclonic and GTCS | Yes | No | No | VPA (750) | Yes |  |
| 21 | | F | 31 | 18 | 18 | 9 | No | Myoclonic seizures | Yes | No | No | LEV (1000) | No |  |
| 22 | | M | 17 | 8 | 14 | 8 | No | Myoclonic and GTCS | Yes | No | No | LEV (1000) | No |  |
| 23 | | F | 48 | 11 | 11 | 12 | Yes | Absences, Myoclonic, GTCS | Yes | No | Yes | VPA (1000), LEV (500) | Yes |  |
| 24 | | M | 61 | 13,7 | 14 | 14 | No | Myoclonic and GTCS | Yes | Yes | Yes | VPA (500)  LEV (1200) | Yes |  |
| 25 | | F | 34 | 11 | 11 | 12 | No | Myoclonic and GTCS | Yes | No | Yes | VPA (1250),  LEV (750) | Yes |  |
| 26 | | M | 24 | 14 | 14 | 14 | No | Myoclonic and GTCS | Yes | Yes | No | VPA (1000) | Yes |  |
| 27 | | F | 29 | 13 | 13 | 14 | No | Myoclonic and GTCS | Yes | Yes | Yes | LEV (1000), PER (8) | No |  |
| 28 | | M | 28 | 8 | 3 | 8 | No | Myoclonic and GTCS | Yes | No | Yes | VPA (1000), TPM (200),  LTG (50) | Yes |  |
| 29 | | M | 62 | 13 | 13 | 13 | No | Myoclonic seizures | Yes | No | No | VPA (1000) | Yes |  |
| 30 | | M | 39 | 12,5 | 12,5 | 13 | No | Absences, Myoclonic, GTCS | Yes | No | Yes | LEV (3000) | No |  |
| 31 | | F | 19 | 12 | 12 | 12,5 | No | Myoclonic and GTCS | Yes | No | No | LEV (2000) | No |  |

**Abbreviations**: CAE= Childhood absence epilepsy;GTCS= generalized tonic-clonic seizures; SEPs= sensory evoked potentials; ASMs= anti-seizure medications; VPA= valproate; LEV= levetiracetam; LTG= lamotrigine; PER= perampanel; ACZ=acetazolamide; TPM= topiramate; mg/d=milligram per day

**Supplementary Table 2:** **Demographic and clinical features of ‘JME’ cohort**

| Patient | Gender | | Age | Age at JME onset | Age at tremor onset | Age at confirmed JME diagnosis | Previous CAE | Seizures during disease course | Postural hand tremor | Giant SEPs | Drug-resistant epilepsy | Ongoing ASMs  (mg/d) | Ongoing VPA |  |
| --- | --- | --- | --- | --- | --- | --- | --- | --- | --- | --- | --- | --- | --- | --- |
|  | | | F | 36 | 16 | - | 17 | No | Myoclonic and GTCS | No | No | No | LEV (750), VPA (1500) | Yes |
|  | | | F | 44 | 8 | - | 11 | No | Myoclonic and GTCS | No | No | No | LEV (1000) | No |
|  | | | F | 36 | 17 | - | 17 | No | Myoclonic and GTCS | No | No | No | LEV (2000) | No |
|  | | | M | 27 | 11 | - | 11 | Yes | Absences, Myoclonic, GTCS | No | No | No | LEV (1000),  VPA (750) | Yes |
|  | | | F | 14 | 13 | - | 14 | No | Absences, Myoclonic, | No | No | No | LEV (2000) | No |
|  | | | F | 24 | 14 | - | 14 | No | Myoclonic and GTCS | No | No | No | LEV (1000), VPA (1000) | Yes |
|  | | | F | 35 | 12 | - | 13 | No | Myoclonic and GTCS | No | No | Yes | PER (8) | No |
|  | | F | 27 | 15 | - | 17 | No | Myoclonic and GTCS | No | No | Yes | LEV (3000), ACZ (150), PER(4), | No |  |
|  | | F | 47 | 14 | - | 15 | Yes | Absences, Myoclonic, GTCS | No | No | No | LTG (400) | No |  |
|  | | F | 30 | 16 | - | 16 | No | Myoclonic and GTCS | No | No | Yes | LEV (1000), LTG (400) | No |  |
|  | | M | 19 | 15 | - | 15 | No | Myoclonic and GTCS | No | No | No | LEV (2000) | No |  |
|  | | M | 25 | 17 | - | 18 | No | Myoclonic and GTCS | No | No | No | VPA (1000) | Yes |  |
|  | | F | 18 | 16 | - | 16 | No | Myoclonic and GTCS | No | No | No | LTG (200) | No |  |
|  | | M | 21 | 16 | - | 17 | No | Myoclonic and GTCS | No | No | No | LEV (1000) | No |  |
|  | | F | 14 | 14 | - | 14 | No | Myoclonic and GTCS | No | No | No | VPA (1000) | Yes |  |
|  | | F | 17 | 12,3 | - | 13 | No | Myoclonic and GTCS | No | Yes | No | LTG (200) | No |  |
|  | | F | 35 | 17 | - | 18 | No | Myoclonic and GTCS | No | No | No | LEV (1250) | No |  |
|  | | F | 17 | 14 | - | 14 | No | Myoclonic and GTCS | No | No | No | LEV (1000) | No |  |
|  | | F | 21 | 18 | - | 19 | Yes | Myoclonic, absences and GTCS | No | No | No | PER (8) | No |  |
|  | | M | 18 | 9 | - | 10 | No | Myoclonic and GTCS | No | No | No | PER (8), TPM (400) | No |  |
|  | | M | 35 | 17 | - | 17 | No | Myoclonic seizures | No | No | No | LEV (500) | No |  |
|  | | F | 36 | 35 | - | 35 | No | Myoclonic and GTCS | No | No | No | LEV (1000) | No |  |
|  | | M | 16 | 8 | - | 8 | No | Myoclonic, GTCS | No | No | Yes | VPA (1500) | Yes |  |
|  | | F | 17 | 13 | - | 13 | No | Myoclonic, GTCS | No | No | No | PER (6) | No |  |
|  | | F | 33 | 14 | - | 14 | No | Myoclonic seizures | No | No | No | LTG (400) | No |  |
|  | | M | 14 | 13 | - | 13 | No | Myoclonic, GTCS | No | No | No | LEV (1000), VPA (500) | Yes |  |
|  | | F | 22 | 10 | - | 11 | No | Myoclonic seizures, | No | No | No | LEV (1000) | No |  |
|  | | F | 17 | 15 | - | 15 | No | Myoclonic, GTCS | No | No | No | LEV (1000) | No |  |
|  | | M | 28 | 12 | - | 12 | No | Myoclonic, GTCS | No | No | No | VPA (750) | Yes |  |
|  | | F | 60 | 13 | - | 13 | No | Myoclonic, GTCS | No | No | No | LEV (1250) | No |  |

**Abbreviations**: CAE= Childhood absence epilepsy;GTCS= generalized tonic-clonic seizures; SEPs= sensory evoked potentials; ASMs= anti-seizure medications; VPA= valproate; LEV= levetiracetam; LTG= lamotrigine; PER= perampanel; ACZ=acetazolamide; TPM= topiramate; mg/d=milligram per day

**6) Supplementary material 3: EEG data**

Each patient underwent standard EEG recording with hyperventilation and photic stimulation according to the latest ILAE recommendations^12^. EEG recordings were classified as either normal or showing interictal epileptiform discharges (IEDs) such as generalized 3–5.5 Hz spike-wave or polyspike-wave activity. We categorized hyperventilation as either absence or presence of evoked IEDs and photostimulation as either normal or showing a “photo-paroxysmal response” (PPR).

At the time of our clinical observation: 30/31(96.8%) people belonging to the JME plus group had IEDs on EEG, compared to 25/30 of the JME cohort [P=0.078, Chi Squared test]. We did not find any significant effect of HV on IEDs in our cohort as our group has previously showed^1^: none of the enrolled subjects had properly HV evoked discharges, while photoparoxymal response (PPR) was found in 8/31(25.8%) and 6/30 (20%) people of JME plus and JME groups, respectively. Thus, we did not find any correlation between these two provocation methods.

We have noticed a good relationship between interictal epileptiform discharges (IEDs) and PPR. In detail, at clinical evaluation 13 out of 14 (92.9%) of the JME individuals with PPR had also IEDs, while only 1 individual with PPR did not show any other IEDs.

References:

1) *Labate, A., Ambrosio, R., Gambardella, A., Sturniolo, M., Pucci, F., & Quattrone, A. (2007). Usefulness of a morning routine EEG recording in patients with juvenile myoclonic epilepsy. Epilepsy research, 77(1), 17–21.* [*https://doi.org/10.1016/j.eplepsyres.2007.07.010*](https://doi.org/10.1016/j.eplepsyres.2007.07.010)
